# Supplementary material for: Structural determinants for GPCR-mediated inhibition of TASK K2P channels by diacylglycerol and its dysfunction in disease
Source: EMBO J. 2026 Feb 25;45(7):2400–12. doi: 10.1038/s44318-026-00710-6 (PMC13043741; doi:10.1038/s44318-026-00710-6)
Supplement: Supplementary file 1 — Table EV1 [file 44318_2026_710_MOESM1_ESM.docx]

**Table EV1**

Values for GqPCR inhibition and single channel *P_o_* for the mutations shown in Fig. 1E.

|  | **GqPCR inhibition (%)** | | ***P_o_*** | |
| --- | --- | --- | --- | --- |
| **Mutation** | **Mean** | **SEM** | **Mean** | **S.E.M** |
| **WT** | 55.31791 | 3.94712 | 0.012 | 0.002 |
| **H98A** | 52.14568 | 3.37074 | 0.012 | 0.002 |
| **Q126E** | 20.74442 | 1.36913 | 0.2 | 0.03 |
| **F125A** | 33.69677 | 2.6548 | 0.033 | 0.023 |
| **F125S** | 15.70827 | 1.26412 | 0.24 | 0.09 |
| **Q126N** | 34.84849 | 3.58943 | 0.021 | 0.005 |
| **R131H** | 7.05866 | 2.23994 | 0.27 | 0.02 |
| **G129A** | 43.7433 | 3.45456 | 0.05 | 0.02 |
| **G129N** | 3.26835 | 2.35972 | 0.42 | 0.01 |
| **G129S** | 38.06134 | 2.76244 | 0.12 | 0.02 |
| **G129D** | 20.31303 | 2.25802 | 0.39 | 0.02 |
| **G129E** | 9.13373 | 1.14078 | 0.68 | 0.07 |
| **G129Q** | 7.98621 | 0.80181 | 0.48 | 0.05 |
| **F125T** | 9.59519 | 1.61699 | 0.169 | 0.03 |
| **F125V** | 27.27258 | 2.67483 | 0.068 | 0.017 |
