# Supplementary material for: Structural determinants for GPCR-mediated inhibition of TASK K2P channels by diacylglycerol and its dysfunction in disease
Source: EMBO J. 2026 Feb 25;45(7):2400–12. doi: 10.1038/s44318-026-00710-6 (PMC13043741; doi:10.1038/s44318-026-00710-6)
Supplement: Supplementary file 2 — Table EV2 [file 44318_2026_710_MOESM2_ESM.docx]

**Table EV2**

Mean lifetimes (τ) and relative areas (*a*) of open states, closed states and burst durations of the probability density functions fitted to the data in Fig. 4.

|  | **τ (ms) (control)** | **τ (ms) 1 μM DiC8** | ***a* (control)** | ***a* (1 μM DiC8)** |
| --- | --- | --- | --- | --- |
| **Open states** | 0.65 | 0.35 | 0.24 | 0.88 |
|  | 1.29 | 0.63 | 0.76 | 0.12 |
| **Closed states** | 0.15 | 0.04 | 0.75 | 0.12 |
|  | 0.26 | 0.15 | 0.18 | 0.18 |
|  | 1.23 | 1.1 | 0.02 | 0.04 |
|  | 10.5 | 23 | 0.008 | 0.22 |
|  | 41 | 56 | 0.036 | 0.44 |
|  | 620 | 330 | 0.0003 | 0.003 |
| **Bursts** | 0.10 | 0.41 | 0.04 | 0.67 |
|  | 1.6 | 0.86 | 0.35 | 0.23 |
|  | 24.5 | - | 0.61 | - |
