# Supplementary material for: Structural determinants for GPCR-mediated inhibition of TASK K2P channels by diacylglycerol and its dysfunction in disease
Source: EMBO J. 2026 Feb 25;45(7):2400–12. doi: 10.1038/s44318-026-00710-6 (PMC13043741; doi:10.1038/s44318-026-00710-6)
Supplement: Supplementary file 12 — Expanded View Figures [file 44318_2026_710_MOESM12_ESM.pdf]

Expanded View Figures

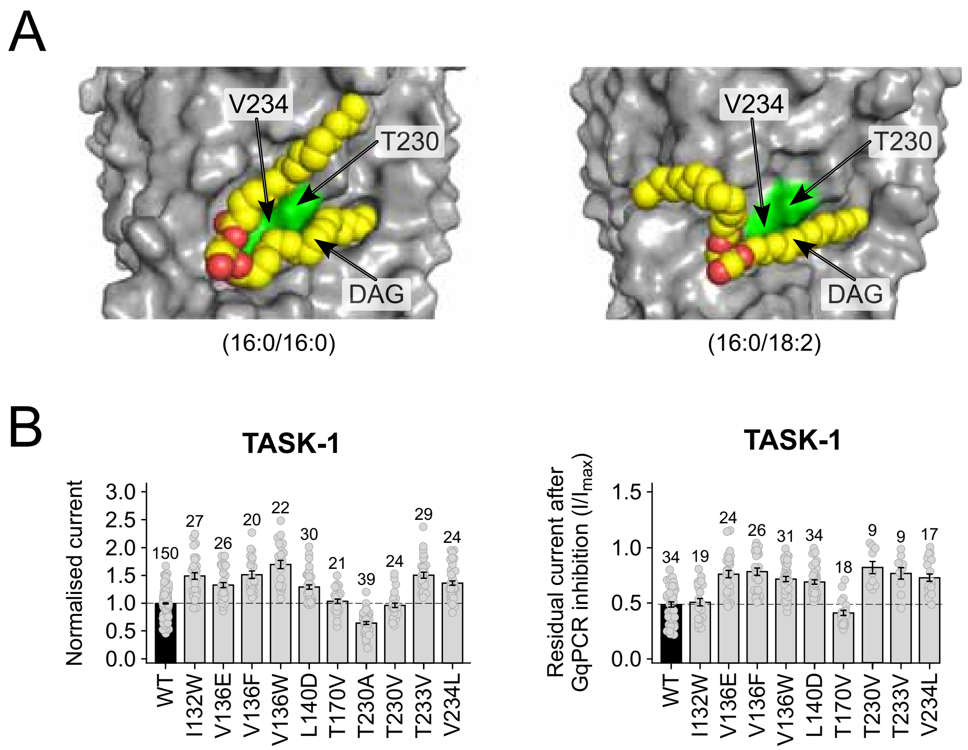

Figure EV1. DAG sensitivity of TASK channels.

(**A**) Less frequent poses of the acyl chains are also found for DAG in the groove between M2, M3 and M4 (see also Fig. 5A). Residues T230 and V234 are shown in green. (**B**) Normalised whole-cell current values and relative GqPCR sensitivity for mutations within this groove. Data information: In (**B**), column bars display mean values with standard error (standard deviation/root(*n*)) based on the number of indicated experiments (*n*; number of individual oocytes). WT effects are highlighted as dashed lines. Source data are available online for this figure.

 © The Author(s)

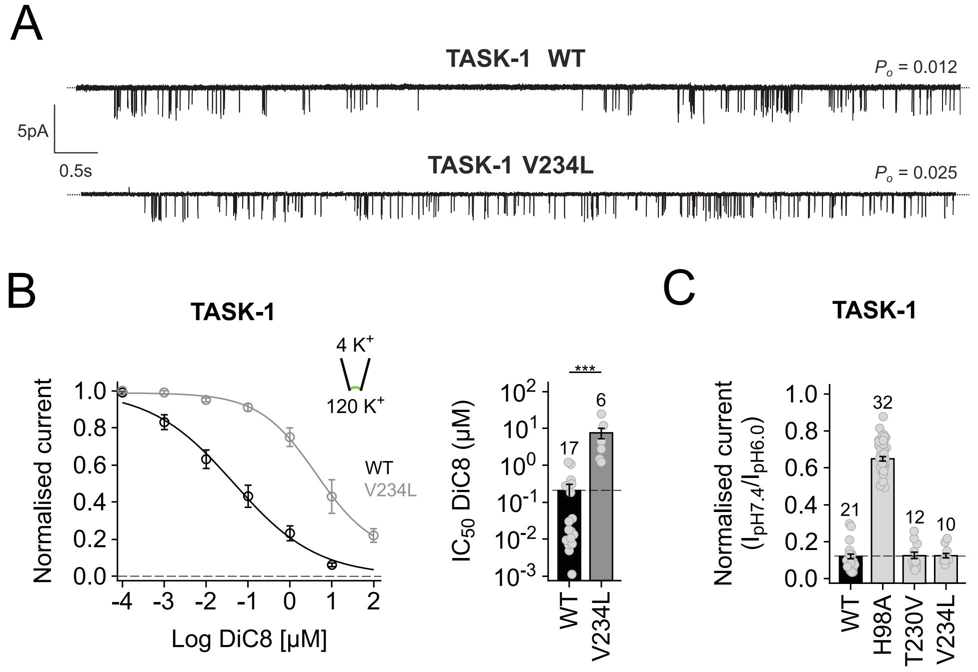

Figure EV2. Molecular determinants of DAG interaction with TASK channels.

(**A**) Single-channel recordings for WT and V234L mutant TASK-1 channels. (**B**) This mutation only increases P_o around twofold yet decreases DiC8 inhibition >100-fold as measured in excised patches. Left: Dose-response curve showing the marked reduction in DiC8 inhibition for V234L compared to WT TASK-1. Right: Reduced IC_{50} values for DiC8 inhibition of V234L (***$p < 0.001$). (**C**) T230V and V234L mutant channels retain their sensitivity to inhibition by pH_e. The reduced sensitivity of the H98A pH sensor mutation is shown as a control. Data information: In (**B**), circles display mean values with standard error (standard deviation/root(*n*)) based on the number of individual experiments (*n*; excised patches). Column bars display mean values with standard error (standard deviation/root(*n*)) based on the number of indicated experiments (*n*; number of excised patches). Significance of changes were determined using the Wilcoxon rank test. ***$p = 5.53*10^{-5}$. In (**C**), column bars display mean values with standard error (standard deviation/root(*n*)) based on the number of indicated experiments (*n*; number of individual oocytes). In both, the WT effect is highlighted as a dashed line. Source data are available online for this figure.
